# A cost effectiveness study of integrated care in health services delivery: a diabetes program in Australia

**DOI:** 10.1186/1472-6963-8-205

**Published:** 2008-10-06

**Authors:** Ian S McRae, James RG Butler, Beverly M Sibthorpe, Warwick Ruscoe, Jill Snow, Dhigna Rubiano, Karen L Gardner

**Affiliations:** 1 Australian Centre for Economic Research on Health, The Australian National University, Canberra, Australia; 2 Australian Primary Health Care Research Institute, The Australian National University, Canberra, Australia; 3 The Menzies School of Health Research, Darwin, Australia; 4 Southern Highlands Division of General Practice, Bowral, Australia

## Abstract

**Background:**

Type 2 diabetes is rapidly growing as a proportion of the disease burden in Australia as elsewhere. This study addresses the cost effectiveness of an integrated approach to assisting general practitioners (GPs) with diabetes management. This approach uses a centralized database of clinical data of an Australian Division of General Practice (a network of GPs) to co-ordinate care according to national guidelines.

**Methods:**

Long term outcomes for patients in the program were derived using clinical parameters after 5 years of program participation, and the United Kingdom Prospective Diabetes Study (UKPDS) Outcomes Model, to project outcomes for 40 years from the time of diagnosis and from 5 years post-diagnosis. Cost information was obtained from a range of sources. While program costs are directly available, and costs of complications can be estimated from the UKPDS model, other costs are estimated by comparing costs in the Division with average costs across the state or the nation. The outcome and cost measures are used derive incremental cost-effectiveness ratios.

**Results:**

The clinical data show that the program is effective in the short term, with improvement or no statistical difference in most clinical measures over 5 years. Average HbA1c levels increased by less than expected over the 5 year period. While the program is estimated to generate treatment cost savings, overall net costs are positive. However, the program led to projected improvements in expected life years and Quality Adjusted Life Expectancy (QALE), with incremental cost effectiveness ratios of $A8,106 per life-year saved and $A9,730 per year of QALE gained.

**Conclusions:**

The combination of an established model of diabetes progression and generally available data has provided an opportunity to establish robust methods of testing the cost effectiveness of a program for which a formal control group was not available. Based on this methodology, integrated health care delivery provided by a network of GPs improved health outcomes of type 2 diabetics with acceptable cost effectiveness, which suggests that similar outcomes may be obtained elsewhere.

## Background

There has been a plethora of research on the nature of diabetes and the response of the disease to various treatment regimes, which has led to a broad agreement on the nature of appropriate care for people with diabetes [1,2]. This agreement has led to the preparation of guidelines for the management of diabetes in many countries including Australia [3]. There is strong evidence that guidelines-based care can improve patient outcomes [4] particularly when supported by central computerized systems for patient tracking and provision of feedback to physicians [5].

While the cost-effectiveness of many diabetes treatments has been assessed (e.g. [2,6,7]) there has been little assessment of guideline implementation. Recent studies from the Netherlands [8,9] found implementation of diabetes guidelines to be cost effective, with the level of cost effectiveness varying between different aspects of care. Other studies have addressed particular components of guidelines [10-12], or the value of guidelines in diabetes prevention [13]. Each adopts, to a greater or lesser degree, the approach of using primary data to measure dissemination and implementation effects, and models to estimate long term treatment expenses and outcomes. This approach has been recommended in a systematic review of the effectiveness and efficiency of guideline implementation [14].

There are no published studies on the cost effectiveness of guideline implementation in Australia, though one study [15] assessed the costs of implementation of multi-disciplinary systematic care. Other Australian studies address the cost impact of diabetes care in Indigenous Australian communities [16] or draw on overseas results to model the potential cost effectiveness of prevention programs [17,18].

This study addresses the cost effectiveness of a program designed to integrate diabetes care and to improve guideline implementation in an Australian Division of General Practice. Divisions are regional networks of general practices which, among other things, provide a platform for regional approaches to the management of type 2 diabetes in primary health care through the implementation of guidelines-based programs. The purpose of the study is to examine whether a Division-based program, which co-ordinates information and provision of care, improves outcomes for diabetic patients and is cost effective.

There are currently 119 Divisions in Australia which vary in their geographic coverage, location, population and numbers of general practices. They have a range of roles including: maintaining and improving the standards of general practice in a region; coordinating care between GPs and other service providers; improving communication between general practice, hospitals, medical specialists and community health services and; taking an active role in the continuum of education from undergraduate through to postgraduate vocational training in general practice [19,20].

A recent report on the value of the Divisions network found little objective evidence examining the effects of Divisions [21]. The few studies that have been undertaken rely on process measures rather than health outcome measures to investigate the effectiveness of Divisions. This study, using clinical measures and a simulation model, is able to estimate outcome measures for the diabetes program in one Division. While the results are specific to the Division chosen, they provide an indication of the potential benefits of the use of this approach more widely across the Divisions network, and also illustrate the potential for applying this methodology more widely to assess existing programs.

As the study is observational, data are drawn from a range of sources to assess the overall costs and benefits. The perspective taken is of the health system as a whole, including all costs on whomsoever they fall. This encompasses the national tax-funded health insurance scheme which may be considered statutory insurance, voluntary health insurance, and patient out-of-pocket costs.

## Method

### The intervention

The Southern Highlands Division of General Practice (SHDGP) in New South Wales (NSW) implemented a diabetes program in 1995. All GPs in the SHDGP participate in the program. The GPs remain the case managers for their patients, and the program facilitates case management by provision of information and education to the GPs and by direct service provision to patients referred by the GPs. The Division funds and arranges diabetes education programs, dietitian services and an exercise program, and arranges access to podiatry services.

The core of the program is the centralized database of diabetic patients which is regularly updated with clinical information from the general practices. This database, which includes information on care provision as well as clinical indicators such as HbA1c measures, is used to send recall reminders to GPs, to provide regular audit reports to GPs on their adherence to guidelines, and to provide regular and ad hoc clinical alerts. In particular, the Division identifies patients who may be at risk of developing complications, and reports on them to their GPs.

The centralized database has been adapted for the diabetes program from a patient information database designed to manage information on cardiac and diabetic patients (the CarDiab database [22]). While there are gaps in the database where clinicians have failed to report the results of consultations, or patients have failed to attend for consultations or tests, the quality of data used is high. All pathology data are provided electronically from the pathology laboratories, and demographic and other clinical data are provided directly by the GPs to the Division, either paper-based or electronically. The analysis undertaken in this study draws on information from this centralized database and a range of other sources as outlined below.

The SHDGP is a rural division with the majority of the population living in three small towns. Its catchment population at the 2001 Census was 48,400 with 16% aged over 65 and 33% under 25. The population is above the median for socio-economic status as measured by the Socio-Economic Index for Australia (SEIFA) prepared by the Australian Bureau of Statistics [23]. The Division has 50 GP members in 16 practices and a relatively low level of GP services compared to the national average (3.7 GP services per head of population compared with 4.7 for Australia as a whole) [24].

### Methods

A cost-effectiveness analysis of the Southern Highlands Division Diabetes Program is undertaken using a decision-analytic approach. As with many recent studies [13,6,25] a simulation model is used to predict longer term patient outcomes and complications for patients in the program. To our knowledge there is no diabetes simulation model available based on Australian data. The United Kingdom Prospective Diabetes Study (UKPDS) Outcome Model [19,26] used in this study is based on ten years data from the UKPDS. This model predicts the costs and the outcomes for patients from their time of diagnosis with type 2 diabetes until death. Simulations are based on a probabilistic discrete-time illness-death model which has been validated within the UKPDS populations. The UKPDS model is designed to capture the association between different types of complications at an individual patient level[27]. It includes the major complications of diabetes, but not other problems such as ulceration and hyper/hypoglycemia [26].

Projections from the UKPDS outcomes model are determined by observed clinical measures (including HbA1c, blood pressure, BMI and cholesterol measures) both at diagnosis and where available over a post-diagnosis period. The model was used in this study without modification to simulate the progression of diabetes and its complications from time of diagnosis to death, and from a time point which is 5 years after initial participation in the program to death. The model simulates the progression of diabetes under "conventional treatment" within the UKPDS study. "Conventional treatment" is prescribed in the UKPDS as clinic attendance every three months and dietary advice, aimed to maintain fasting plasma glucose below 15 mmol/L without symptoms of hyperglycemia [2]. Conventional treatment in Australian practice will be much more varied. The implications of this potential difference are likely to be small, as the issue of interest is the difference between projections from diagnosis and projections after 5 years in the program. Where the estimated impact of the program on the costs of complications as derived from the model can be compared with estimates derived from other sources, results are of a similar order.

It is common practice for models to draw on data from countries other than those which are the subject of their study. The CORE model for example [28], used data from the UKPDS, data from the Framingham Study and other data sources in United States, data from Sweden and information from the international DIGAMI trial.

A sample of patients enrolled in the program was selected as outlined below and the average annual incremental cost of managing them in the diabetes program estimated. These costs encompassed both the direct costs of managing the program, and any incremental costs or savings flowing from changes in care for program participants. Four categories of costs were included: the cost of the SHDGP diabetes program; the primary care costs costs arising from adherence to the guidelines for management of diabetes; the cost of pharmaceuticals; and the cost of inpatient hospital services.

Differences in costs in three of these categories (primary care services provided in compliance with guidelines, pharmaceutical treatment, and hospital treatment) cannot be directly measured from the Division database. However, it is possible to estimate these costs by comparing costs in the SHDGP with costs for NSW or for Australia as a whole. Cost estimates for managing complications from the UKPDS Outcomes Model are used to provide estimates of the costs of hospital treatment for comparison with the direct estimates available from Australian information.

In summary, data from SHDGP patients are used to estimate program impacts on the life years and quality adjusted life expectancy of these patients, and to estimate the costs of diabetes caused complications. These cost estimates contribute to the estimated impact of the program on hospital costs, while the program impacts on life years and QALE are used to estimate cost effectiveness.

To convert the estimated annual program costs and the related costs and savings to a 40-year time horizon, the average annual estimates are assumed to be the same each year over the 40-year time frame of the analysis. The cost streams are discounted at five per cent per annum. Although the modelling incorporates both deaths and increased costs per capita due to complications as patients age, it does not address per capita cost changes for pharmaceutical costs or costs of GP compliance with guidelines. If the single year overall treatment savings followed the pattern of the costs of complications, the 40 year total estimate would be only 5 per cent different to the value estimated by assuming constant costs. The dominant factor in the overall costs is the direct cost of the program, which will not increase with patient ageing except at the margin (e.g. dietician costs), but will decline with the deaths of program participants. Allowance for the effect of these deaths on program costs reduces the long term costs by substantially more than any plausible increase in treatment cost effects, but a fixed value approach was taken to ensure that results were conservative.

The five per cent discount rate follows the standards used in Australia in assessing pharmaceuticals and medical services for public funding [29,30]. This level of discounting is consistent with German, Swiss and French discounting rates [18], although for the United Kingdom rates of 3.5% are used [25] and rates of 1.5% for clinical outcomes and 6% for costs are quoted [18], and for the Netherlands 3% is used [12]. In the sensitivity analyses, a rate of 0% is tested, but as 5% is at the higher end of international discounting rates no higher value is used.

The health outcomes for the selected patients are estimated over a 40-year period from diagnosis, using the model of the long-term sequelae of patients with type 2 diabetes in the UKPDS Outcomes model. The measures used are the changes in years of life expectancy and in QALE. The impact on utility of different diabetes related complications used in the model to estimate QALE is based on a study of a UKPDS participants in 1997 [31].

### Patient sample

Using National Health Survey data [32], the number of identified type 2 diabetics in the SHDGP in 2005 was expected to be 1,525. Overall the program had contact with around 85% of the expected total number of identified diabetics in the region in 2004, although for 220 of the known patients there were no clinical data.

While there were 1,087 type 2 diabetic patients included on the database at some time over the program, the number included in the analysis was considerably less than this as:

• The UKPDS model is designed to commence at time of diagnosis, so patients who joined the program post diagnosis were excluded;

• Clinical advice suggested that patients should participate for around 5 years so that behavioural changes in particular have stabilized (as the program had been running only 10 years, this requirement significantly reduced the available sample);

• Data on all clinical items used in the model were required at both diagnosis and after 5 years. Similar systems report only 50% to 60% of patients with an HbA1c score recorded in any year [1], and in this study recording of clinical results was similarly incomplete (to enhance the sample size, if clinical data after 5 years were not available, data after 4 years or 6 years were accepted.

As a result of these exclusions, the final sample comprised 74 patients who registered at diagnosis and had complete information both at diagnosis and approximately 5 years after diagnosis.

As this group includes only five-year survivors, six additional patients who were randomly selected from among those deceased within five years of joining the program were added to the initial sample. Six patients were added as eight per cent of the total patient group who registered at or near diagnosis died during the following five years.

Gaps in the SHDGP data combined with the requirements of the UKPDS model resulted in a sample which could have introduced bias. However, as shown in Table 1, the characteristics of all patients on enrolment were similar to those of the study sample. Significant differences which exist are in the expected directions. Table 1 also shows the program has been effective in improving the status of the sample over 5 years. Most clinical measures (e.g. cholesterol) have improved or are stable, and the increase in average HbA1c is less than would have been expected over 5 years under normal treatment [27].

**Table 1:** Comparison of characteristics of type 2 diabetes patients in the SHDGP Diabetes Program and patients included in the model^(a)^

Characteristics	All patients at registration	Patients included in the model		
		
		All	5 Year Survivors	
			
		At registration^(c)^	At registration	After 5 Years^(d)^
Gender (% female)	46.0	51.3	50.0	50.0
Average age at diagnosis (years)	59.2	62.3*	61.1	66.1
Average duration of diabetes (years)	4.1	0.1	0.1	5.1
Average HbA1c	7.4	6.9*	6.9	7.2
Average systolic blood pressure	135.9	135.1	135.1	132.9
Average lipid cholesterol level	5.4	6.1*	6.2	5.1**
Average HDL level cholesterol	1.3	1.2	1.2	1.3
Average BMI	31.1	31.7	31.9	32.0
% current smokers of those with known smoking^(b)^	15.0	20.0	21.6	21.6
				
Number of patients	1,372	80	74	74

**Note: **(a) There are different numbers of missing values for each characteristic for "All patients". There are 80 observations for all characteristics for patients included in the model.

(b) Smoking changes rarely reported.

(c) Asterisks show significantly different averages between the population and the group of 80 patients included in the modelling

▪ Age at diagnosis: as the patients in the model join the program at diagnosis they have lower average age at registration than the overall population of patients;

▪ Average HbA1c is higher for the population overall as they have a longer duration of diabetes

▪ Lipid cholesterol is lower for the population overall as they have been accessing lipid lowering drugs for longer.

(d) Double asterisk shows the significant differences between the patients included in the model at registration and 5 years later

▪ The only statistically significant difference is improved cholesterol, again almost certainly due to lipid lowering drugs.

**Source: **HDGP CarDiab database

### Health outcomes

Life years and QALE were estimated over 40 years from diagnosis using the UKPDS Outcome model. Outcomes were first estimated from diagnosis with no constraint, and secondly estimated with the constraints of actual clinical measures after five years of program participation. These two sets of projections gave estimates of life years and QALE saved by program participants.

### Southern Highlands Division of General Practice Diabetes Program costs

Division administrative costs, including the costs of employing the program manager/diabetes educator, data entry, general administration and IT costs, and the costs of patient access to dietetic services and the exercise program were provided by the Division. The details of these costs are shown at Additional file 1.

Administrative costs to practices included maintenance of records and transfer of data to the Division, and the management of patient recall. These costs were estimated by the Division based on consultation with the participating practices and are also shown at Additional file 1.

### Costs of compliance with guidelines

The cost of GP compliance with guidelines, and the flow on costs to pathology testing, ophthalmology etc, were based on data from the Service Incentives Payments (SIP) scheme [33]. SIP payments are made to registered GPs on completion of a 12 month cycle of guidelines based care for a diabetic patient. Claims were made for 31% of known diabetic patients in the SHDGP compared to 20% across Australia as a whole [33]. These percentages were combined with estimates of average national costs per diabetic patient-year for out of hospital services [34] (excluding pharmaceutical costs), and assumptions on relative costs of compliant and non-compliant treatment, to provide estimates of costs of guideline compliance (see Additional file 1).

### Pharmaceutical costs

A comparison of prescribing rates for oral antidiabetic agents in the SHDGP with national rates, using publicly available data from the Pharmaceutical Benefits Scheme (PBS), was used to estimate differences in pharmaceutical costs attributable to the program[35]. Details again are shown in Additional file 1. As it is not possible to divide insulin prescribing between type 1 and type 2 diabetic patients, the cost of insulin has not been included. On the other hand, these data do not include patient co-payments, which would lead to an under-estimate of any savings.

### Costs of hospital services

Three estimates of cost savings were obtained for hospital services. One estimate was based on the difference between rates and costs of hospitalization for patients with primary ICD-10 diagnosis codes 10–14 (diabetes) residing in the Southern Highlands and in New South Wales overall. These estimates were based on data provided by NSW Department of Health. As these are diabetes-specific codes, they will not include all complications of diabetes. The cost per patient-year for these two groups ($A255 and $A341 respectively) are well below the Australian estimate of around $A600 per patient year [34] for diabetes-related hospitalization. However, the actual hospitalization data provide an indication of the level of savings in hospital costs for program participants.

The second estimate applied the percentage reduction in the costs of treating complications attributed to the program from the UKPDS model to an estimate of the average annual cost of hospitalizations for type 2 diabetic patients in Australia [34]. The third approach used actual dollar values of savings estimated by the UKPDS Outcomes model. The three approaches provided estimates of a similar order of magnitude, as shown at Additional file 1.

## Results

Details of all costings are shown at Additional file 1. The annual overall program cost per diabetic patient is estimated to be $A196, including approximately $A100 of direct Divisional costs, $A10 dietician costs, and $A6 costs of an exercise program. The costs were calculated by averaging total annual costs across 1,087 active type 2 diabetic patients.

Costs to practices depend on whether electronic or paper systems were used, and were estimated to be between $A30 and $A80 per patient per year. The higher level of practice costs is consistent with previous research [15], which found ongoing average practice costs (in 2005 prices) were $A5,798 per practice compared with the average cost per practice in this study of $A6,535 (using the $A80 per patient cost). Total program costs therefore range from $A146 to $A196 per patient per year, with the higher value used as the base estimate.

The estimated costs of additional compliance with diabetes treatment guidelines for the SHDGP relative to the rest of Australia are shown in detail in Additional file 1. Depending on assumptions regarding the relative average costs of guideline compliant and non-compliant treatment, the estimated additional costs of compliance in the SHDGP range from $10 to $90 per patient per year, with a value of $A50 being used in the cost effectiveness calculations. This figure is consistent with the costs of compliance being around twice the costs of non-compliance. If patients following guidelines attended GPs four times per year, and other patients attended annually, with testing following similar ratios, an average cost ratio of four would be possible. Many patients who do not fully comply with guidelines, however, still attend a GP relatively frequently (if only to obtain the relevant medicines). A range from 1.25 to 4.00 in relative costs was therefore used.

Estimated costs for treatment of complications over 40 years derived from the UKPDS model, together with 95% confidence intervals, are shown in Table 2. In proportionate terms, the program achieves an estimated 7.4% reduction in treatment costs for complications. Applying this percentage to the average annual cost of hospitalization of diabetic patients in Australia [34] gives an estimated saving of $44 per patient-year between the baseline and the program group.

**Table 2:** Costs for diabetes-related complications and health outcomes from the UKPDS Outcomes Model using the Australian patient sample from the SHDGP Diabetes Program

	Treatment cost^(a)(b)^		Difference^(b)^	
		
	No program	Program	Absolute	%
	£8,325	£7,708	-£617	-7.4
	(£7,076 – £9,574)	(£6,444 – £8,972)	(-£2,394 – +£1,160)	

	Health outcome^(c)^		Difference^(c)^	
		
	No program	Program	Absolute	%

Life-years	10.89	11.25	0.36	3.3
	(10.52 – 11.26)	(10.87 – 11.63)	(-0.17 – +0.89)	
QALE	8.25	8.54	0.30	3.6
	(7.98 – 8.51)	(8.27 – 8.81)	(-0.08 – +0.68)	

(a) Treatment cost for diabetes-related complications from the UKPDS model using clinical data from the Australian patient sample

(b) 95% confidence intervals in parentheses. Confidence interval for difference calculated as though independent estimates. These costs are health care costs associated with diabetes-related complications. They do not include ongoing care costs other than for complications. Costs reflect health care utilisation in the UK in the year 2000. Discount rate = 5% per annum

(c) 95% confidence intervals in parentheses. Confidence interval for difference calculated as though independent estimates. Discount rate = 5% per annum.

**Source: **SHDGP Diabetes Program; UKPDS Outcomes Model [26]

Table 2 also shows the absolute value of cost savings per patient over 40 years to be £617, which is equivalent to $A1,470 in 2005 prices, and to $A81.50 per patient-year savings (assuming a constant cost over time). The use of the proportionate reduction in costs applied to Australian estimates of the annual costs of hospitalization is preferred as the cost structures and boundaries of "costs of complications" will not be the same in the UK and in Australia, while the proportionate effect of the program on the costs of complications is likely to be same as the proportionate effect on Australian hospital costs.

Comparing average hospital costs across the relevant ICD-10 codes (codes 10–14) per known diabetic patient in SHDGP with the average across New South Wales provided an estimated net saving of $86 per patient year, consistent with the estimated savings derived from the UKPDS Outcomes model. To be conservative, the figure of $44 saving was used in the cost effectiveness calculations, while the higher $86 figure was used in the sensitivity analyses.

The average cost to the PBS per patient year of prescribing of oral anti-diabetic drugs in the SHDGP is approximately $40 less than the national figure. While the estimated $40 savings in prescribing is used to derive the estimated cost effectiveness, a lower bound estimate of zero savings is used in the sensitivity analyses.

Table 3 summarizes the estimated impact of the program on hospitalization, antidiabetic prescribing, and guideline compliance, with an aggregate estimate of overall treatment costs savings of $A34 per patient-year against the cost of the diabetes program of $196 per patient year. The net cost of the program is positive, even when the most optimistic figures are taken for both treatment effects and program costs. The overall net cost estimates range from $30 per patient-year to $242 per patient-year as shown in Table 4.

**Table 3:** Treatment cost savings from SHDGP Diabetes Program (AUD 2005)

	Cost saving per patient-year(a)	Range for sensitivity testing
Hospitalization costs^(b)^	-44	-86 to -44
Antidiabetic prescribing^(c)^	-40	-40 to 0
Guideline compliance^(d)^	+50	+10 to +90
		
Total	-34	-116 to +46

(a) Negative sign indicates cost saving

(b) AIHW estimate health expenditure on diabetes per patient-year of $A1,469, with 37% for hospital services. Updating to 2005 prices gives an estimated expenditure of $A601 per patient-year on hospital services. Estimated savings are obtained by applying the percentage reduction in costs of treating complications from the UKPDS model (7.4% – see Table 2). The larger saving of $A86 per patient-year used in sensitivity testing is the difference between the cost of hospital episodes with ICD-10 codes 10–14 for SHDGP ($A255 per patient-year) and for NSW ($A341). Data provided by NSW Health.

(c) The difference between the annual cost to the PBS of prescription antidiabetic agents per type 2 diabetic patient in SHDGP ($A80.95) and nationally ($A121.50). Reflects a difference in the prescribing rate of 5.1 scripts per person in SHDGP and 7.7 nationally.

(d) The increase in treatment cost due to guideline compliance of $A50 is an indicative figure based on the average annual cost per diabetic patient of out-of-hospital medical services and other professional services of $A454 in 2005 prices and the difference between the proportion of known diabetic patients giving rise to SIP claims in SHDGP (31%) and nationally (20%).

Source: SHDGP Diabetes Program; [34], p.1 and Table 2; [33,35]

**Table 4:** Results of cost effectiveness analysis for the SHDGP Diabetes Program^(a)^

	Base case	Lower bound	Upper bound
Cost of SHDGP Diabetes Program per patient-year (Additional file 1)	$196	$146	$196
Treatment cost savings per patient-year (Table 3)	-$34	-$116	+$46
Net cost per patient-year	$162	$30	$242
Discounted net cost per patient over 40 years^(b)^	$2,919	$540	$4,360
Life-years saved (Table 2)	0.36	0.36	0.36
Increase in QALE (Table 2)	0.30	0.30	0.30
Cost per life-year saved	$8,108	$1,502	$12,111
Cost per year increase in QALE	$9,730	$1,802	$14,533

**Note: **(a) All dollar values are expressed in Australian dollars, 2005 prices.

(b) Assumes net cost per patient-year is the same each year for 40 years. Discount rate = 5% per annum.

**Source: **SHDGP Diabetes Program; UKPDS Outcomes Model [26]; [34], p.1 and Table 1; [33,35]

The health outcomes derived from the UKPDS Outcome Model are shown in Table 2. The program is estimated to achieve an increase in discounted life expectancy of 0.36 years and an increase in discounted QALE of 0.30 years. While neither of these estimated differences is significantly different from zero using 95% two sided tests (with standard errors of the difference conservatively calculated), the probability that there is a zero or negative effect is small for both measures (life years: p = 0.09; QALE: p = 0.06).

Table 4 presents the results of the cost-effectiveness analysis. The net program cost per patient over 40 years (discounted at five per cent) is estimated to be $A2,919. The resulting cost-effectiveness ratios are $8,108 per life year saved, and $9,730 per year increase in QALE.

The cost-effectiveness ratios resulting from this study are within the generally acceptable limits applied to health care interventions. The only publicly available assessment of limits in Australia shows that drugs are unlikely to be recommended for listing on the PBS if they cost over $A76,000 per life year and unlikely to be rejected if they cost less than $A42,000 per life year (1998–99 values) [36]. A range of thresholds have been proposed or have been implicitly used in other countries to evaluate the reasonableness of costs per quality adjusted life year, and almost all are over $A32,000 in 2005 prices [37].

## Sensitivity analyses

Three aspects of this study are addressed in assessing the robustness of this main conclusion. Firstly, the five per cent discount rate, while the Australian standard, is at the high end of international standards. Secondly the effect on the conclusion of the range of estimates from the different cost components is tested. Finally, the potential variability in the gain in life years or QALE is considered.

If no discounting was applied to costs or outcomes, the improvement in expected life years and QALE from program participation increases (from 3.3 per cent to 3.6 per cent, and from 3.6 per cent to 4.5 per cent respectively), and the percentage savings diminish (from 7.4 per cent to 6.3 per cent). Recalculating Table 4 with no discounting, the estimated cost per QALE rises from $9,730 to $11,172, with a similar increase in the cost per life year. These estimates remain well within the range of acceptable costs.

As shown in Table 4, the estimated costs per life year or per QALE using the maximum values of all cost components, are $12,111 and $14,533 respectively. While approximately 50 per cent above the base case, these figures also remain well within acceptable bounds.

**Figure 1:**
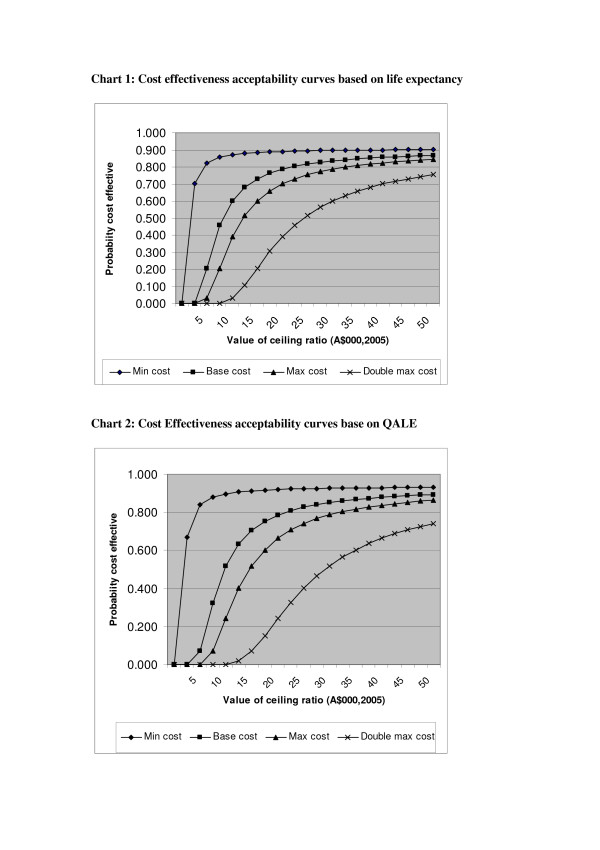
Cost effectiveness acceptability curves

Finally, the estimated improvements in health outcomes are also subject to estimation error. This variability can be displayed by use of cost effectiveness acceptability curves (see for example [25,38]). Please see Figure 1 for charts 1 and 2 below show the probability that the cost per year of life expectancy gained, or the cost per year of quality adjusted life expectancy gained, is below any given value of a ceiling ratio (a willingness to pay). Minimum and maximum costs are shown as well as the best estimate. As all cost estimates are positive, a decision maker who was only prepared to accept this program with zero net cost would not accept it, so the curves all show zero probability of acceptance at zero willingness to pay.

However, the curves show that if a figure of $A42,000 [36] is used as the acceptable limit (although it was seven years old at the time of the study), using the base costing there is a better than 85 per cent chance that the incremental cost effectiveness ratio is less than this limit for both life years saved and increased QALE.

Using the maximum estimated cost of the program there is a better than 80 per cent chance the ratio is less than $42,000. Even if the upper limit of the total net costs is doubled, the probabilities of the program being within the $42,000 willingness-to-pay limit are 71% and 68% for life years and QALE respectively. The conclusions are therefore very robust to both the variation of the estimated outcomes and the known limitations in the costing methods.

## Discussion

Despite the estimated improvement in health outcomes and reduced hospital costs, the study estimates an increase in the overall net costs from the program. This is consistent with other programs which improve diabetes outcomes[25,8,9]. While outcomes can be improved at relatively low cost, few of the approaches reduce costs overall.

It has proved necessary to draw on a wide range of data to estimate the cost impact of the SHDGP diabetes program. While there are limitations in the methods and the data, the use of a range of cost estimates provides considerable robustness to the overall conclusion to be drawn regarding the cost effectiveness of the program.

With any program addressing long term chronic diseases like diabetes, it is necessary to use modelling approaches to estimate long term outcomes and costs [14]. As long term Australian data are not available, the use of a model based on other data was inevitable. This follows the same logic as other studies [28] which draw on many sources for their model components.

Some costs and savings are estimated by comparing costs in the SHDGP with those in the state of NSW, or in Australia as a whole. These estimates could be biased as the SHDGP is not the only division to implement a diabetes program. However, a 2002 survey covering 80 per cent of divisions with a diabetes program [39] found only approximately 25,000 of the (self reported) 554,000 diabetic patients in 2001 [40] were covered by electronic register recall systems. Given this low coverage, it is unlikely that the other programs significantly influenced the comparative cost estimates in this study.

Policy-makers need to know whether to continue to support and fund organizationally based programs which attempt to integrate care and encourage use of guidelines, but have been put in place without formal control groups. The approach taken here provides a methodology which gives robust answers to questions regarding cost effectiveness, after accommodating the limitations in the data.

The SHDGP diabetes program would appear to have made a cost effective contribution to the wellbeing of the diabetes patient in the SHDGP (although with positive net cost), and as such provides a potential model to be considered in other Divisions. A number of initiatives introduced for GPs in Australia in recent times, like the SHDGP diabetes program, have had as their objective the improvement of care coordination and achievement of more integrated care (for example the inclusion of items in the Australian Medicare Benefits Schedule which provide incentives for GPs to take part in multi-disciplinary care planning, and the incentive payments for GPs who follow guidelines in care of diabetes and asthma). In this respect, the US and Australian health care systems may be heading towards a common end point in the longer term (i.e. integrated care) although arriving at that end point by different routes.

## Conclusion

This study faces the problems inherent in attempting to assess an existing program, with limits to direct data collection and no formal control group. It has been shown, however, that reasonable and robust estimates of cost effectiveness of the diabetes management program can be derived using clinical data from a central database and cost data taken from a range of sources. The results show that a program using a centralized computer-based register, and providing some centralized services, is highly likely to be cost effective although at a positive net cost.

This does not suggest that all programs undertaken by all Divisions will be cost-effective. It does however demonstrate both that it is possible to draw robust conclusions about such programs if the net for data collection is cast suitably wide, and that integrated care for diabetic patients through enhanced GP networks can provide value for money.

## Abbreviations

ADGP: Australian Divisions of General Practice (National council of Divisions of General Practice); AIHW: Australian Institute of Health and Welfare; BP: Blood pressure; CHF: Congestive heart failure; FFS: Fee for service; GP: general practitioner; ICD-10: International Classification of Diseases – version 10; LE: Quality adjusted life expectancy; MI: Myocardial infarction; SBO: State Based Organisation (State level council of Divisions of General Practice); SEIFA: Socio-Economic Index for Australia; SHDGP: Southern Highlands Division of General Practice; SIP: Service incentive payment; UKPDS: United Kingdom Prospective Diabetes Study.

## Competing interests

Dr Warwick Ruscoe and Ms Jill Snow declare that they are employees of the Southern Highlands Division of General Practice. All other authors declare that they have no competing interests.

## Authors' contributions

All authors contributed to the study design. WR and JS facilitated the compilation of the program data. DR and KG undertook background literature research, IM and DR undertook data analysis and drafted the paper, and JB and BS critically revised the paper. All authors have read and approved this version of the manuscript.

## Pre-publication history

The pre-publication history for this paper can be accessed here:



## Supplementary Material

Additional file 1 Attachment 1 Click here for file
