# Supplementary material for: A cost effectiveness study of integrated care in health services delivery: a diabetes program in Australia
Source: BMC Health Serv Res. 2008 Oct 6;8:205. doi: 10.1186/1472-6963-8-205 (PMC2577097; doi:10.1186/1472-6963-8-205)
Supplement: Additional file 1 — Attachment 1 [file 1472-6963-8-205-S1.doc]

**ATTACHMENT 1: SUMMARY OF COSTS**

| **Source** | **Costing derivation** | **Costs per patient year^1^** |
| --- | --- | --- |
| Divisional costs | Total cost $120,000 per annum^2^  In 2004, 1394 active diabetic patients, 1087 were type 2 diabetics. $100 is used as indicative figure. | 100 |
| Dietitian | Total cost $15,000 per annum^2^, $10 used as indicative figure | 10 |
| Exercise program | Approx $6,000 per year for diabetic component of program^2^  $6 used as indicative figure | 6 |
| Practice costs of register management | Estimated per patient per year: - electronic practice register $20, non-electronic $100;  25% of patients covered by electronic systems^3^  Implies $80 per patient per year  Alternate figures suggest around $30 per patient per year^3^.  For sensitivity testing use range $30 to $80 per patient per year | 80 |
| *Total Program costs* | Cost estimates range from $146 to $196 per patient per year | *196* |
| Guideline compliance | Percent of known diabetic patient with SIP claims for full guideline compliance: 31% for SHDGP, 20% for Australia as a whole^4^  Average national costs of out of hospital medical services per diabetic patient per year $454^5^  Ratio of costs of treatment when compliant versus treatment which does not comply expected to be in range 1.25 to 4, giving difference in average cost of between $A12 and $A94  Range $A10 to $A90 for sensitivity testing, $A50 base estimate | 50 |
| Prescribing | Pharmaceutical Benefits claims for oral antidiabetic agents per type 2 diabetic patient in 2004-05^6^  SHDGP 5.1 scripts per patient at a cost of $80.95  Australia as a whole: 7.7 scripts per patient and $121.50  Base estimate savings of $A40. $A0 used for sensitivity testing. | -40 |

| Hospitalization | Approach 1: UKPDS modelling estimated savings from complications of 7.4%. Applying to estimated average $601 per patient per year hospitalization costs in Australia [34] gives an estimated saving of $A44 per patient per year  Approach 2 UKPDS modelling estimated savings for complications of £617 per patient over 40 years. Converting to $A and to a single year figure (assuming constant costs per year) gives $81  Approach 3 Hospital costs^7^ for directly diabetes related ICD-10 codes (excluding hospital costs which may be diabetes related such as strokes, heart attacks etc) show $A255 per patient per year for SHDGP, $A341 for NSW, giving a saving of $86 per patient per year. | -44 |
| --- | --- | --- |
| Total | Total costs range from $A34 to $A242 per year. | $162 |

- 1. Costs in $A, 2005
  2. Information provided by Division
  3. Information provided by Division based on consultation with practices
  4. Data extracted from Medicare Australia website [41]
  5. From Australian Institute of Health and Welfare, data updated to 2005 prices [34]
  6. Data extracted from Medicare Australia website [42]
  7. Data provided by the NSW Department of Health for 2004
